# Determination of five flavonoids in different parts of *Fordia cauliflora* by ultra performance liquid chromatography/triple-quadrupole mass spectrometry and chemical comparison with the root of *Millettia pulchra* var. *laxior*

**DOI:** 10.1186/1752-153X-7-126

**Published:** 2013-07-19

**Authors:** Lanlan Fan, Yazhou Zhang, Renbin Huang, Shanding Qin, Tao Yi, Feng Xu, Yina Tang, Xiaosheng Qu, Hubiao Chen, Jianhua Miao

**Affiliations:** 1 Guangxi Botanical Garden of Medicinal Plants, Nanning, China; 2 School of Chinese Medicine, Hong Kong Baptist University, Hong Kong Special Administrative Region, China; 3 School of Pharmaceutical Science, Guangxi Medical University, Nanning, China; 4 School of Pharmaceutical Sciences, Peking University, Beijing, China

**Keywords:** *Fordia cauliflora*, *Millettia pulchra* var. *laxior*, Fordiae Cauliforae Radix, Millettiae Pulchrae Radix, UPLC-QqQ-MS, UPLC-DAD, Determination, Identification, Ethnic drug

## Abstract

**Background:**

The root of *Fordia cauliflora* Hemsl (FC) has long been used in southern China for the treatment of rheumatism, bruises, dementia in children, and valetudinarianism. However, sometimes it is mixed with other parts. And it has always been confused with the root of *Millettia pulchra* (Benth.) Kurz var. *laxior* (Dunn) Z. Wei (MP) by the local people. The chemical differences between the two ethnic drugs are not clear until now. The aim of this study is to develop a precise and accurate method to characterize and quantify multiple chemical components of FC, which is helpful for the quality evaluation and identification of FC.

**Results:**

A method coupling ultra performance liquid chromatography (UPLC) with triple-quadrupole mass spectrometry (QqQ-MS) was first developed for simultaneous determination of five flavonoids in different parts of FC and the root of MP, based on a UPLC-diode array detection (DAD) fingerprint method. All calibration curves showed good linearity (R^2^>0.99) within test ranges. The overall LOD and LOQ were lower than 2.5 ng/mL and 5.0 ng/mL, respectively. The RSDs for intra- and inter-day of five analytes were less than 2.83% and 3.04%, respectively. Recovery studies for the quantified compounds were found to be within the range 93.6-99.8% with RSD less than 5.73%. The results suggest that the root, traditionally used medicinal part, yields the highest flavanoid content in FC. Pachycarin A, 3′,4′-dimethoxy(2′′,3′′:7,8) furanoflavone, karanjachromene and isoderricin A can be used to differentiate between FC and MP samples.

**Conclusions:**

The present method is specific, precise and reliable, and is suitable for characterizing and quantifying multiple chemical components of FC.

## Background

The root of *Fordia cauliflora* Hemsl (FC), Fordiae cauliflorae Radix, from the Leguminosae family[1-3], known as “Shuiluosan”, has been used for the treatment of rheumatism, bruises, dementia in children, valetudinarianism and so on by the Zhuang and Yao people in Guangxi Zhuang Autonomous Region of China for over five hundred years. Pharmacological studies have shown that it improves short- and long-term memory and acquired memory disorder of mice [4,5], and that it has anti-aging [6], anti-inflammatory [7], hepato-protective [8], and antioxidative [8] effects. However, the root is difficult to obtain than other parts, such as stem and leaves, and thus, sometimes they have been mixed with other parts.

The root of *Millettia pulchra* (Benth.) Kurz var. *laxior* (Dunn) Z. Wei (MP), Millettiae Pulchrae Radix, known as “Daluosan” [3,9], is also used for the treatment of rheumatism, bruises, dementia in children, valetudinarianism and so on [3,10]. The local people always confused FC with MP, because they share similar therapeutic functions and are both called “Luosan” in the locality. By the way, the major active compounds of FC and MP are flavonoids, such as furonaflavones, pyranoflavones and chalcones [9-11]. The only reported major constituent in common, namely karanjin, possesses potential pesticidal, anti-inflammatory effects [11]*et al*. However, the chemical composition differences between FC and MP are still not clear. Therefore, it is necessary to develop an effective method to evaluate FC, and distinguish it from MP.

The modern technologies, UV [12], TLC [13,14], TLC-FI [15], and HPLC [16,17], have been utilized for the quality control of FC and MP. However, no fingerprint analysis of the two medicines has been reported yet. Nowadays, ultra-performance liquid chromatography coupled with mass spectrometry technology has been found to be a superior method for the quality evaluation of herbal medicines due to the high resolution and detection sensitivity [18-21]. The compounds in a mixture can be efficiently separated by UPLC, and can be characterized by MS. Accordingly, in the present study, an ultra performance liquid chromatography coupled with triple-quadrupole mass spectrometry (UPLC-QqQ-MS) was developed, based on a UPLC-diode array detector (DAD) fingerprint method, for the determination of five key flavonoids in three different parts of FC samples from different geological areas, and then compared with the root of MP. The validation results revealed that the developed method is highly efficient and reliable, and hence suitable for quantitative analysis and identification of FC samples.

## Experimental

### Materials, chemicals and reagents

Fifteen samples of *Fordia cauliflora* Hemsl (FC1-FC15) and two samples of *Millettia pulchra* (Benth.) Kurz var. *laxior* (Dunn) Z. Wei (MP1-MP2) were collected from Guangxi Zhuang Autonomous Region of China. FC1-FC3 were collected from Guangxi Botanical Garden of Medicinal Plants, MP1 and MP2 were cultivated in Lingshan, and the rest of the samples were obtained from the wild. Identity of the samples was confirmed by the authors, and voucher specimens were deposited in the School of Chinese Medicine, Hong Kong Baptist University.

**Figure 1:**
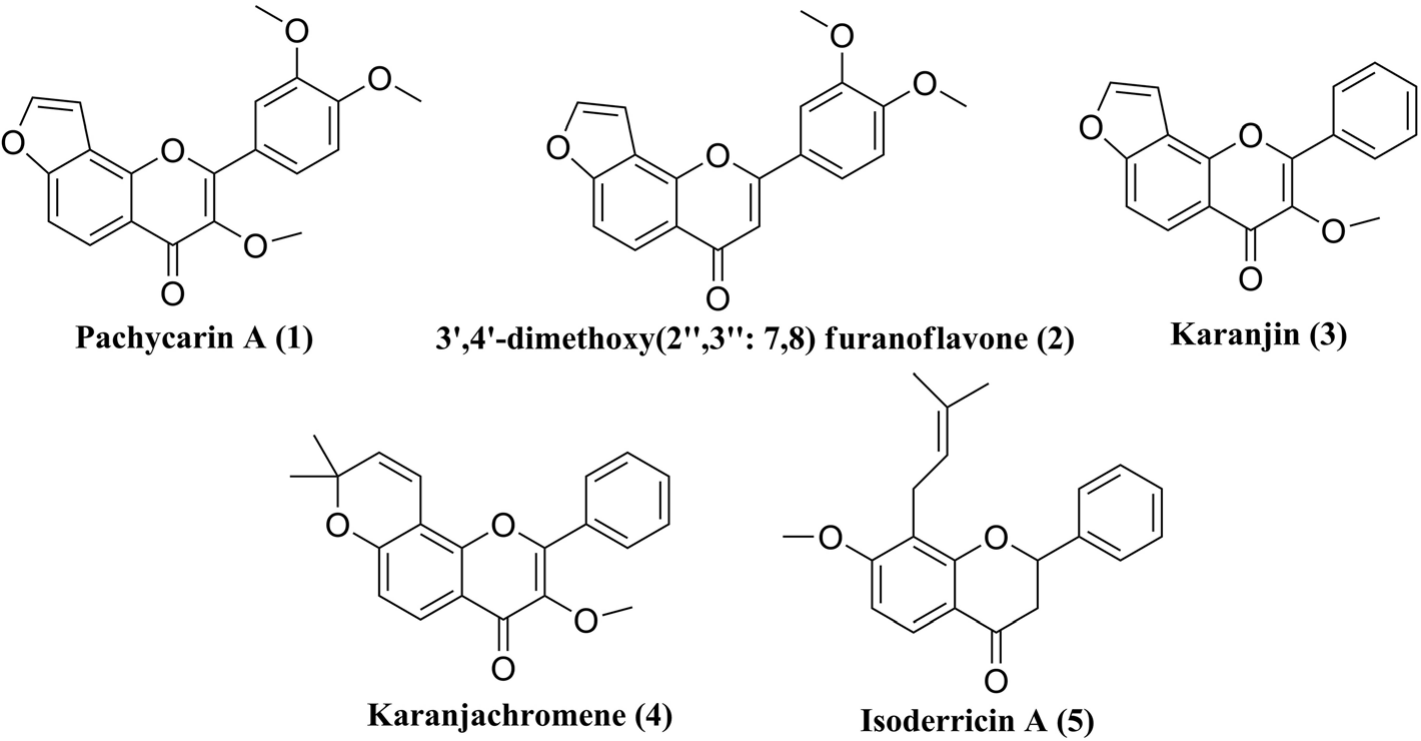
Chemical structures of the five analytes.

Pachycarin A, 3′,4′-dimethoxy(2′′,3′′:7,8) furanoflavone, karanjin, karanjachromene and isoderricin A were separated and purified in our laboratory (98%, determined by HPLC). The chemical structures of the five compounds are shown in Figure 1.

Analytical grade methanol and chromatographic grade acetonitrile were purchased from Labscan (Bangkok, Thailand), and chromatographic grade formic acid was purchased from Fluka (Buchs, Switzerland). Deionized water was obtained from a Milli-Q water purification system (Millipore, Bedford, MA, USA).

### Preparation of sample solutions

All samples were ground into powder, and passed through a 40-mesh sieve. The sample powder (0.25 g) was extracted with 10 mL of methanol by means of ultrasonication at room temperature for 0.5 h. These operations were repeated once, and the residue was washed with 4 mL of methanol. Total extracts were combined in a 25-mL volumetric flask, which was filled to the calibration mark with extraction solvent. The extracts were then filtered through a syringe filter (0.22 μm, Alltech, Beerfield, IL, USA) before used.

### Ultra performance liquid chromatography-diode array detector (UPLC-DAD) fingerprint analysis

A Waters Acquity™ ultra-performance liquid chromatography (UPLC) system (Waters Corp., Milford, USA) consisting of a binary pump, autosampler, thermostated column compartment and diode array detector (DAD), was used for fingerprint analysis. The chromatographic separation was carried out on a Waters ACQUITY UPLC® HSS column (100 × 2.1 mm i.d., 1.8 μm, Waters Corp.) with a VanGuard™ pre-column (HSS, C_18_, 1.8 μm, 2.1 mm × 5 mm). The mobile phase consisted of 0.1% formic acid (FA) in water (A) and 0.1% FA in acetonitrile (B) using a gradient program of 25-40% (B) in 0–4 min, 40-66% in 4–23 min. The solvent flow rate was 0.35 mL/min, the column temperature was set to 40°C and the detection wavelength was 258 nm. The filter samples were diluted 10 times with methanol. An aliquot of 5 μL solution was injected for analysis.

### Ultra performance liquid chromatography-triple quadrupole mass spectrometry (UPLC-QqQ-MS)

An Agilent 6460 Triple Quadrupole LC/MS system (Agilent Technologies, the USA) was used. The chromatographic separation was carried out on a Waters ACQUITY UPLC ® HSS column (100 × 2.1 mm i.d., 1.8 μm, Waters Corp.) with a VanGuard™ pre-column (HSS, C_18_, 1.8 μm, 2.1 mm × 5 mm). The mobile phase consisted of 0.1% FA in water (A) and 0.1% FA in acetonitrile (B) using a gradient program of 35-85% (B) in 0–11 min. The solvent flow rate was 0.35 mL/min, the column temperature was set to 40°C. The filtered samples were diluted 20 times with methanol. An aliquot of 1.0 μL solution was injected for analysis.

The column effluent was directly introduced into a triple quadrupole mass detector operated in a positive ESI mode. Nitrogen was used as both the drying and sheath gases and the collision gas. The ESI source parameters were as follows: gas temperature, 300°C; gas flow, 7 L/min; nebulizer gas pressure, 45 psi; sheath gas temperature, 350°C; sheath gas flow, 8 L/min; capillary voltage, 3.5 kV. The fragmentor voltage was 130 V for all compounds. The collision energy was selected for each compound individually, and ranged from 21 to 81 eV. Samples were analyzed by UPLC-QqQ-MS in the multiple reaction monitoring (MRM) mode to maximize sensitivity. Characteristic transitions (precursor ion→product ion) are shown in Table 1. Data analysis was performed with Agilent Mass Hunter Qualitative Analysis B.04.00 Software (Agilent Technologies, the USA).

**Table 1:** MRM transition parameters of five analytes (Positive ESI mode)

**Peak**	***R*t (min)**	**Compounds**	**MRM transition**	**Fragmentation (V)**	**Collision energy (eV)**
1.	5.04	Pachycarin A	353>323	130	29
			353>295	130	33
2.	5.60	3′,4′-dimethoxy(2′′,3′′:7,8) furanoflavones	323>293	130	33
3.	6.17	Karanjin	293>277	130	33
			293>89	130	81
4.	8.46	Karanjachromene	335>305	130	29
			335>187	130	49
5.	9.66	Isoderricin A	323>105	130	25
			323>163	130	21

### Method validation

Each standard was dissolved in methanol and then mixed as stock solution at the concentration of 1 mg/mL, and stored in the refrigerator. The working solutions were prepared by appropriate dilution, and the resulting concentration ranges are listed in Table 2. The calibration curve was established by plotting the peak area against the concentrations of the standards with linear regression analysis. The limit of detection (LOD) and limit of quantification (LOQ) for each analyte were determined at an S/N of about 3 and 10, respectively. Intra- and inter-day variations were chosen to determine the precision of the developed method. For the intra-day variation test, three levels of the mixed standards solution was analyzed for six replicates (n=6) within one day, while for the inter-day variations test, the three levels was examined in duplicates for consecutive 3 days (n=6). The repeatability of the method was determined by analyzing one FC samples for six replicates and represented as RSD. The recovery was performed by adding three levels of known amount of stock solution into a certain amount of FC samples. Two replicates of each sample were extracted and analyzed.

**Table 2:** Linearity curves, LOD and LOQ for five analytes

**Compounds**	**Linear equations**	**Range (ng/mL)**	***R***^**2**^	**LOD (ng/mL)**	**LOQ (ng/mL)**
Pachycarin A	y=768.5x+7174.2	2.5-2500	0.9989	0.2	1.0
3′,4′-dimethoxy(2′′,3′′:7,8) furanoflavones	y=15702.1x+161577.2	2.5-2500	0.9991	0.2	1.0
Karanjin	y=5434.7x+95172.3	2.5-2500	0.9985	0.1	0.5
Karanjachromene	y=7224.7x+37031.0	2.5-2500	0.9996	0.1	0.5
Isoderricin A	y=1832.1x+64281.6	5.0-10000	0.9996	2.5	5.0

## Results and discussion

### Optimization of analysis conditions

The extraction solvents initially tested were methanol, 80% methanol, 50% methanol and ethanol. The results revealed that extraction with absolute methanol produced the highest yield for the desired analytes. Thus, methanol was chosen as the extraction solvent. Extraction times and cycles were further optimized, and the results demonstrated that exhaustive extraction could be achieved when 0.2 g of FC powder was extracted with 10 mL of methanol with two sonication cycles of 0.5 h.

The conditions for chromatographic analysis including the type of column and column temperature were optimized. Mobile phase gradients were compared on an HSS C_18_ column and a BEH C_18_ column at different temperatures. The results showed that satisfactory separation could best be obtained by eluting FC samples on an HSS C_18_ column at 40°C using a linear gradient of 0.1% formic acid (FA) in acetonitrile and 0.1% FA in water within 11 min.

A wavelength of 258 nm was chosen to monitor the constituents in UPLC-DAD after comparing the chromatograms of the FC and MP samples recorded at wavelengths within 190–500 nm. It was found that the analysis time of UPLC-QqQ-MS could be shortened from 22 min to 11 min without loss of accuracy; thus the shorter period was chosen to save time and solvent.

### UPLC-DAD fingerprint

**Figure 2:**
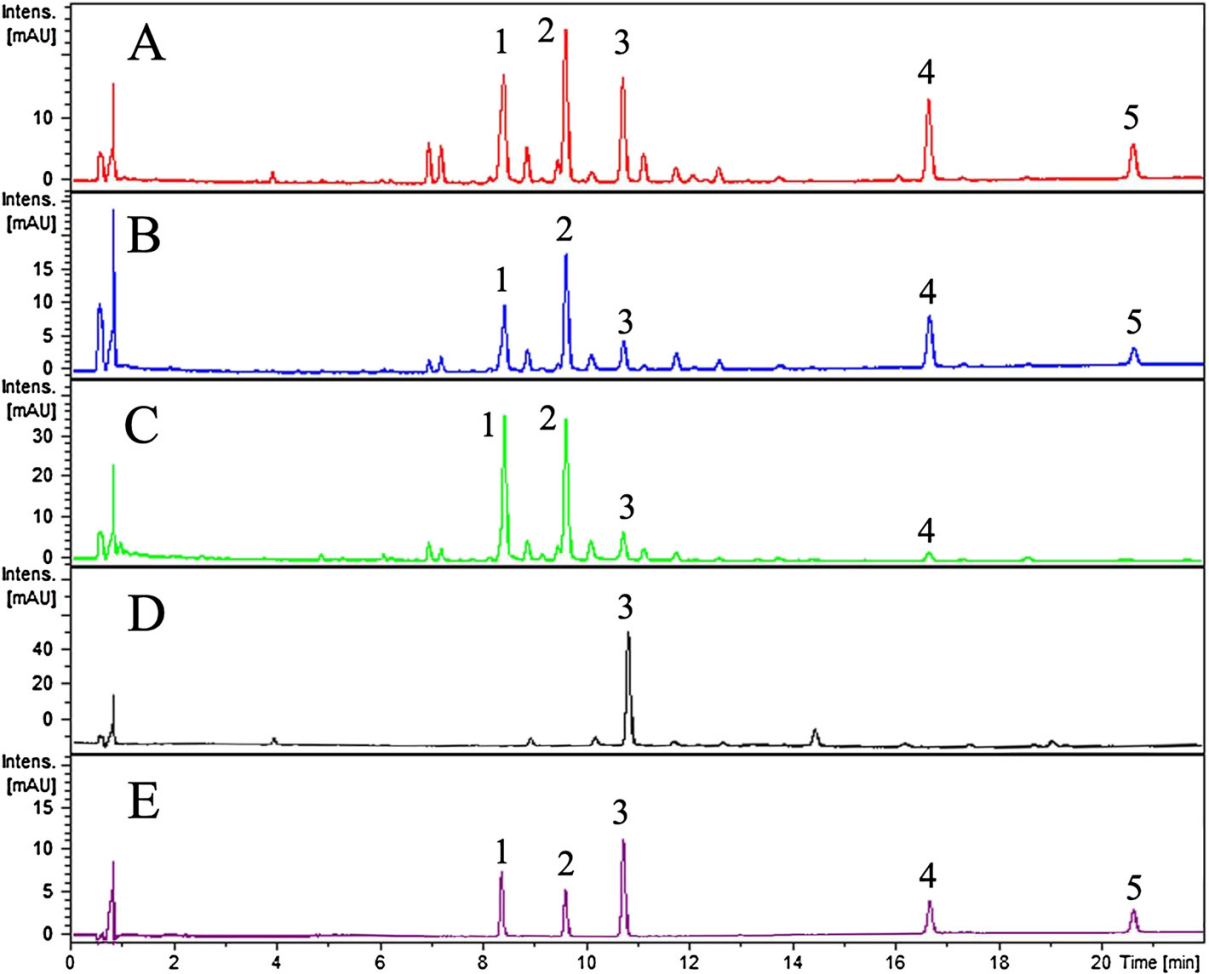
**Representative UPLC-DAD fingerprint chromatograms of roots of *Fordia cauliflora *(A), stems of *F. cauliflora *(B), leaves of *F. cauliflora *(C), roots of *Millettia pulchra *var. *laxior *(D), and reference compounds (E).** Peak identification: 1. Pachycarin A, 2. 3′,4′-dimethoxy(2′′,3′′:7,8) furanoflavone, 3. Karanjin, 4. Karanjachromene, and 5. Isoderricin A.

The representative UPLC-DAD fingerprint chromatograms of roots, stems and leaves of *Fordia cauliflora* (FC), and roots of *Millettia pulchra* (Benth.) Kurz var. *laxior* (Dunn) Z. Wei (MP) are shown in Figure 2. This is the first report about the fingerprint of FC and MP. With respect to FC, pachycarin A, 3′,4′-dimethoxy(2′′,3′′:7,8) furanoflavone, karanjin, karanjachromene and isoderricin A are the major peaks in the roots. The chemical composition of the stems was similar to the roots, while some peaks were absent in the leaves, such as isoderricin A. However, fewer peaks were detected in the roots of MP. Karanjin showed the highest peak in MP, and was seen in both FC and MP.

### MS characterization of the five flavonoids

The positive ionization mode was selected for quantification tests. To enhance the selectivity and sensitivity of the MS detection, multiple reaction monitoring (MRM) [18] was employed. To determine the best MRM parameters for the five flavonoids, pachycarin A (1), 3′,4′-dimethoxy(2′′,3′′:7,8) furano flavone (2), karanjin (3), karanjachromene (4) and isoderricin A (5), their MS and MS/MS spectra were measured by the infusion of each flavone, one by one.

Both the precursor ions ([M+H]^+^) of (2) and (5), *m/z* 323, were selected as the precursor ions. However, the product ion of (2) was *m/z* 293, while for (5) was *m/z* 105 and 163. The details are listed in Table 1.

In order to promote the formation of quasi-molecular ions [M+H]^+^ in MS analysis, 0.1% formic acid was used in the mobile phase.

### Method validation of UPLC-QqQ-MS

#### Specificity

**Figure 3:**
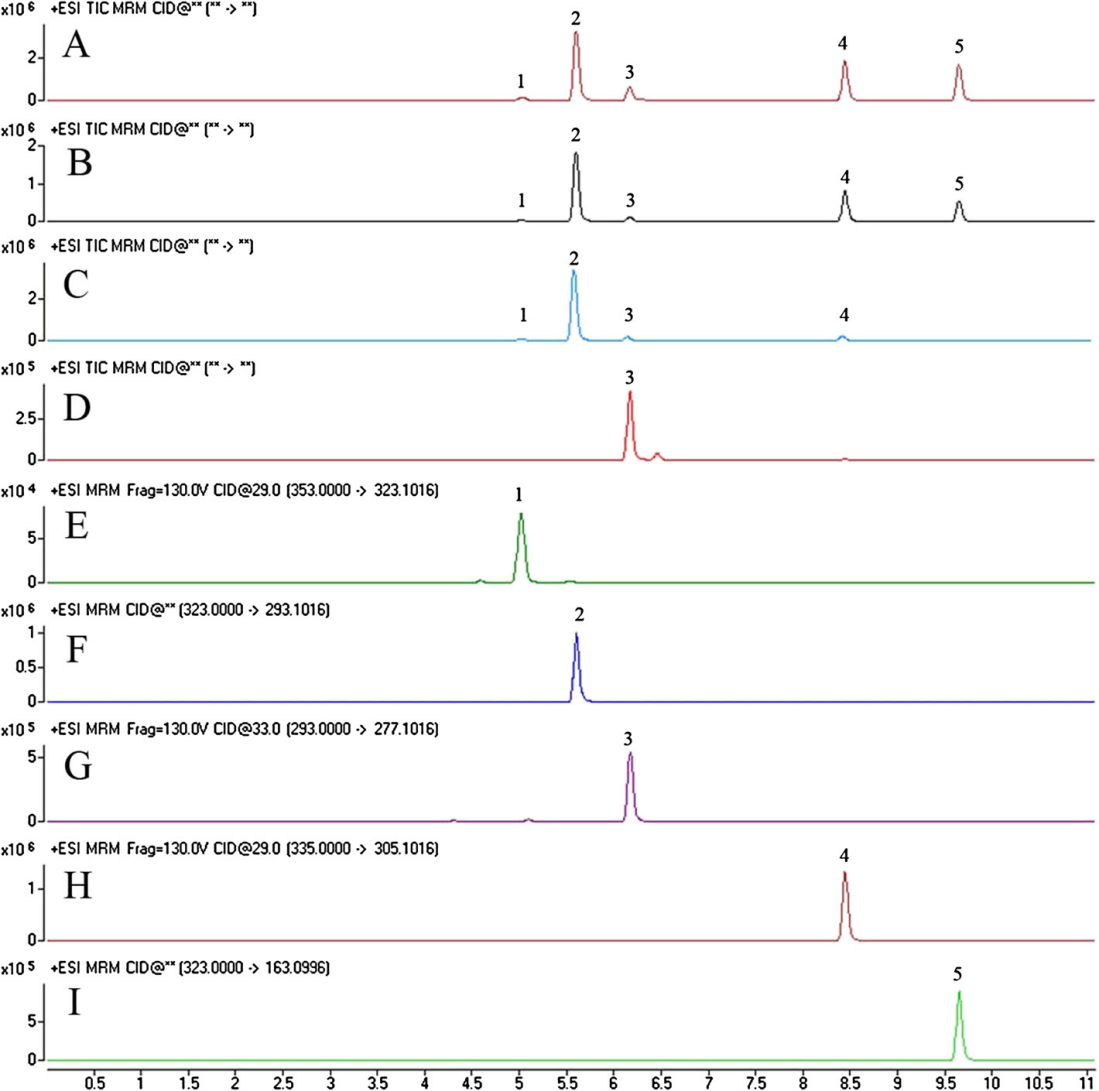
**Representative MRM chromatograms of roots of *F. cauliflora *(A), stems of *F. cauliflora *(B), leaves of *F. cauliflora *(C), roots of *M. pulchra *var. *laxior *(D), and the five analytes (E-I) by UPLC-QqQ-MS.** Peak identification: 1. Pachycarin A, 2. 3′,4′-dimethoxy(2′′,3′′:7,8) furanoflavone, 3. Karanjin, 4. Karanjachromene, and 5. Isoderricin A.

The representative MRM chromatograms of the three parts of *F. cauliflora* (FC) and root of *M. pulchra* (Benth.) Kurz var. *laxior* (Dunn) Z. Wei (MP) are shown in Figure 3. UPLC-QqQ-MS in MRM mode could clearly separate and identify the five compounds, thus saving analysis time. The retention times of pachycarin A, 3′,4′-dimethoxy(2′′,3′′:7,8) furanoflavone, karanjin, karanjachromene and isoderricin A were 5.04, 5.60, 6.17, 8.46 and 9.66 min, respectively. All the peaks of the analytes were detected with excellent resolution.

#### Linearity

The regression equations, correlation coefficients, test ranges, LODs and LOQs are shown in Table 2. The results showed that there was excellent correlation between the ratio of peak area and concentration for each compound within the test ranges. The LODs were 0.2, 0.2, 0.1, 0.1 and 2.5 ng/mL for pachycarin A, 3′,4′-dimethoxy(2′′,3′′:7,8) furanoflavone, karanjin, karanjachromene and isoderricin A, and the LOQs were 1.0, 1.0, 0.5, 0.5 and 5.0 ng/mL, respectively, indicating that this method is sensitive for the quantitative evaluation of the five compounds.

### Precision, repeatability and extraction recovery

The assay precision results are shown in Table 3. The intra- and inter-day precisions (RSD) of these analytes were all less than 2.83% and 3.04%, respectively. The repeatabilities (RSD, n=3) of the five compounds were all less than 3.14%. The mean extraction recoveries and RSD of pachycarin A were 99.8% and 1.27%, of 3′,4′-dimethoxy(2′′,3′′:7,8) furanoflavone were 93.6% and 4.80%, of karanjin were 97.1% and 5.29%, of karanjachromene were 95.4% and 5.73%, and of isoderricin A were 95.5% and 4.68%. The results demonstrated that the values were all within the acceptable range, and that the method was accurate and precise.

**Table 3:** Intra- and Inter-day variation of the investigated compounds

**Compounds**	**Concentration**	**Intra-day RSD**	**Inter-day RSD**
	**(ng/mL)**	**(%, n = 6)**	**(%, n = 6)**
Pachycarin A	1000	1.60	1.67
	500	2.13	2.42
	250	1.89	3.04
3′,4′-dimethoxy(2′′,3′′:7,8) furanoflavones	1000	1.81	2.97
	500	2.26	2.83
	250	1.99	2.50
Karanjin	1000	2.58	2.99
	500	2.83	4.08
	250	2.11	2.07
Karanjachromene	1000	1.15	2.75
	500	2.28	3.11
	250	2.15	2.88
Isoderricin A	1000	1.23	1.41
	500	1.83	2.67
	250	1.47	1.11

### Sample analysis

The developed UPLC-QqQ-MS method was employed for the determination of five flavonoids in three different parts (roots, stems and leaves) of FC samples from five locations, and the roots of MP samples. Typical MRM chromatograms are shown in Figure 3. Table 4 summarizes the contents of the investigated compounds. Several conclusions can be drawn from our results:

**Table 4:** Contents of five compounds in the FC and MP samples

**Species**	**Code**	**Used part**	**Habitats**	**Collection time**	**Contents of five compounds (mg /g, n = 3)**					
					**Pachycarin A (1)**	**3′,4′-dimethoxy(2′′,3′′:7,8) furanoflavone (2)**	**Karanjin (3)**	**Karanjachromene (4)**	**Isoderricin A (5)**	**Total**
*Fordia cauliflora*	FC1	root	Nanning, Guangxi	2011.8.14	0.78 ± 0.04	1.57 ± 0.03	0.85 ± 0.03	1.61 ± 0.05	4.19 ± 0.04	9.00
	FC2	stem	Nanning, Guangxi	2011.8.14	0.09 ± 0.00	0.88 ± 0.01	0.12 ± 0.00	0.71 ± 0.02	1.28 ± 0.01	3.08
	FC3	leaves	Nanning, Guangxi	2011.8.14	0.20 ± 0.00	1.60 ± 0.03	0.20 ± 0.01	0.18 ± 0.01	ND	2.18
	FC4	root	Nanning, Guangxi	2012.7.12	2.69 ± 0.05	0.10 ± 0.00	2.16 ± 0.04	1.75 ± 0.01	3.14 ± 0.03	9.84
	FC5	stem	Nanning, Guangxi	2012.7.12	0.44 ± 0.02	ND	0.22 ± 0.01	0.33 ± 0.01	0.48 ± 0.00	1.47
	FC6	leaves	Nanning, Guangxi	2012.7.12	1.99 ± 0.08	0.01 ± 0.00	0.36 ± 0.01	0.24 ± 0.01	ND	2.60
	FC7	root	Wuming, Guangxi	2012.7.12	0.45 ± 0.02	0.69 ± 0.01	1.90 ± 0.06	0.82 ± 0.03	4.68 ± 0.04	8.54
	FC8	stem	Wuming, Guangxi	2012.7.12	0.05 ± 0.00	0.20 ± 0.01	0.34 ± 0.01	0.27 ± 0.01	0.71 ± 0.03	1.57
	FC9	leaves	Wuming, Guangxi	2012.7.12	0.18 ± 0.01	1.51 ± 0.02	ND	0.22 ± 0.01	ND	1.91
	FC10	root	Pingle, Guangxi	2012.7.10	1.46 ± 0.05	1.32 ± 0.03	2.32 ± 0.04	1.58 ± 0.03	1.17 ± 0.03	7.85
	FC11	stem	Pingle, Guangxi	2012.7.10	0.08 ± 0.00	0.12 ± 0.00	0.04 ± 0.00	0.11 ± 0	0.15 ± 0.01	0.50
	FC12	leaves	Pingle, Guangxi	2012.7.10	0.55 ± 0.01	0.40 ± 0.01	0.01 ± 0.00	0.06 ± 0.00	ND	1.02
	FC13	root	Pingxiang, Guangxi	2012.7.12	2.16 ± 0.06	0.06 ± 0.01	2.49 ± 0.05	1.66 ± 0.03	7.91 ± 0.15	14.28
	FC14	stem	Pingxiang, Guangxi	2012.7.12	0.17 ± 0.01	ND	0.12 ± 0.01	0.14 ± 0	0.38 ± 0.01	0.81
	FC15	leaves	Pingxiang, Guangxi	2012.7.12	2.87 ± 0.03	ND	0.39 ± 0.01	0.28 ± 0.01	ND	3.54
*Millettia pulchra* var. *laxior*	MP1	root	Lingshan, Guangxi	2012.7.12	ND	ND	0.65 ± 0.01	ND	ND	0.65
	MP2	root	Lingshan, Guangxi	2012.7.12	ND	ND	0.50 ± 0.01	ND	ND	0.50

*ND* Not detected.

First of all, the content of each compound varied in different parts of FC, and has their own distribution characteristics. The total content ranges of the five compounds were 8.54-14.28, 0.49-3.07, and 1.02-3.54 mg/g for roots, stems and leaves of FC samples, respectively. The contents of pachycarin A, karanjin, karanjachromene and isoderricin A were highest in the roots, while the content of 3′,4′-dimethoxy(2′′,3′′:7,8) furanoflavone varied; indeed, sometimes it could not be detected. Isoderricin A could not be detected in the leaves, even though it was obtained in the highest content among the five compounds. So the root yields the highest flavanoid content, which is in accordance with the fact that the root is traditionally used for medicinal purposes.

Secondly, only karanjin was detected in both MP and FC, and the other four flavonoids were absent in MP. The contents of karanjin in the two MP samples were 0.50 and 0.65 mg/g, respectively. Thus the other four compounds, pachycarin A, 3′,4′-dimethoxy(2′′,3′′:7,8) furanoflavone, karanjachromene and isoderricin A, can be used to distinguish the two medicinal plants.

Thirdly, the content ranges of pachycarin A, 3′,4′-dimethoxy(2′′,3′′:7,8) furanoflavone, karanjin, karanjachromene and isoderricin A in the roots of FC samples from different habitats were 0.45-2.69, 0.10-1.57, 0.85-2.49, 0.82-1.75, and 1.17-7.91 mg/g, respectively. However, the contents of each compound seem to have no relation with the distribution area. Just to mention, there is only one report regarding the HPLC quantification of karanjin and 3’-methoxykaranjin in FC and MP [16]. Therefore this report is the first one to simultaneously quantify pachycarin A, 3′,4′-dimethoxy(2′′,3′′:7,8) furano flavone, karanjachromene and isoderricin A in FC and MP. However, our results show that the content ranges of karanjin from FC and MP are 0.85-2.49 mg/g and 0.50-0.65 mg/g, respectively, as mentioned above. These data are lower than those reported, in which the content of karanjin from four FC samples was 2.13-4.54 mg/g, and from MP samples was 0.61-1.22 mg/g. This may be due to different harvesting times and habitats.

As we know, flavonoids are the major constituents of FC and MP, and play very important roles in the pharmaceutical activities of FC and MP, such as karanjin as mentioned before, and karanjachromene, which possesses significant antioxidant activity [22]. Our results show that the root yields the highest flavanoid content, so it is reasonable that the medicinal part is the root. The results also suggest that pachycarin A, 3′,4′-dimethoxy(2′′,3′′:7,8) furanoflavone, karanjachromene and isoderricin A can be used to distinguish FC from MP samples.

## Conclusions

A novel UPLC-QqQ-MS method was first developed for the simultaneous analysis of five flavonoids in the root, stem and leaves of *Fordia cauliflora* (FC) based on a first reported UPLC-DAD fingerprint method. The method was also applied to the root of *Millettia pulchra* var. *laxior* (MP) to compare their chemical characteristics. The present hyphenation procedure is highly efficient and reliable, and hence suitable for quantitative analysis of FC samples. The determination results show the root of FC has the highest flavonoid content, and suggest that pachycarin A, 3′,4′-dimethoxy(2′′,3′′:7,8) furanoflavone, karanjachromene and isoderricin A, can be used for the identification of FC and MP samples.

## Abbreviations

UPLC: Ultra performance liquid chromatography; DAD: Diode array detector; QqQ-MS: Triple-quadrupole mass spectrometry.

## Competing interests

The authors declare that they have no competing interests.

## Authors’ contributions

HBC and JHM initiated and all authors designed the study. The sample extraction was conduct by LLF and YZZ. The method developments were conducted by LLF who drafted the manuscript. All authors contributed to data analysis, read and approved the final manuscript.
